# *Plasmodium falciparum*-Infected Erythrocytes Induce Granzyme B by NK Cells through Expression of Host-Hsp70

**DOI:** 10.1371/journal.pone.0033774

**Published:** 2012-03-15

**Authors:** Evelyn Böttger, Gabriele Multhoff, Jürgen F. J. Kun, Meral Esen

**Affiliations:** 1 Institute for Tropical Medicine, Tübingen University, Tübingen, Germany; 2 Department of Radiation Oncology, Klinikum rechts der Isar, Technische Universität München, Munich, Germany; 3 Clinical Cooperation Group - Innate Immunity in Tumor Biology, Helmholtz Zentrum München, Munich, Germany; Federal University of São Paulo, Brazil

## Abstract

In the early immune response to *Plasmodium falciparum*-infected erythrocytes (iRBC), Natural Killer (NK) cells are activated, which suggests an important role in innate anti-parasitic immunity. However, it is not well understood whether NK cells directly recognize iRBC or whether stimulation of NK cells depends mainly on activating signals from accessory cells through cell-to-cell contact or soluble factors. In the present study, we investigated the influence of membrane-bound host Heat shock protein (Hsp) 70 in triggering cytotoxicity of NK cells from malaria-naïve donors or the cell line NK92 against iRBC. Hsp70 and HLA-E membrane expression on iRBC and potential activatory NK cell receptors (NKG2C, CD94) were assessed by flow cytometry and immunoblot. Upon contact with iRBC, Granzyme B (GzmB) production and release was initiated by unstimulated and Hsp70-peptide (TKD) pre-stimulated NK cells, as determined by Western blot, RT-PCR and ELISPOT analysis. Eryptosis of iRBC was determined by Annexin V-staining. Our results suggest that presence of Hsp70 and absence of HLA-E on the membrane of iRBC prompt the infected host cells to become targets for NK cell-mediated cytotoxicity, as evidenced by impaired parasite development. Contact of iRBC with NK cells induced release of GzmB. We propose that following GzmB uptake, iRBC undergo eryptosis via a perforin-independent, GzmB-mediated mechanism. Since NK activity toward iRBC could be specifically enhanced by TKD peptide and abrogated to baseline levels by blocking Hsp70 exposure, we propose TKD as an innovative immunostimulatory agent to be tested as an adjunct to anti-parasitic treatments *in vivo*.

## Introduction

Malaria still remains one of the most devastating infectious diseases world-wide causing ∼655.000 deaths per year, regardless of great efforts in the past years. Immunity against *P. falciparum*, the most dangerous malaria-provoking agent, develops with age over the course of multiple infections and it has been shown that humoral immunity is crucial for protection from severe disease. It is known that Natural Killer (NK) cells play a pivotal role in early innate immune responses by secreting interferon-gamma (IFN-γ) but also via cross-talk and priming of adaptive immunity [1], [2]. Effector functions are triggered by cytokine secretion from and through direct contact with myeloid accessory cells [3], [4]. Originally, NK cells were described as non-specific cytotoxic effector cells killing their target cells without prior sensitization [5], [6]. Nevertheless, during the last years it became apparent that NK cells are more sophisticated. Their function is regulated by numerous inhibiting and activating receptors [7] interacting with a set of different ligands, e.g. stress proteins and MICA/B [8]. Furthermore, it is well-known that they become activated by cells missing MHC class-I molecules on their cell surface [9]. Interestingly, mature erythrocytes do not express MHC, but usually are not eliminated by NK cells. Therefore, either additional signals triggering NK cell-mediated cytotoxicity towards infected erythrocytes (iRBC) must exist or erythrocytes express further inhibiting ligands. Since erythrocytes are host cells for *P. falciparum* and are crucial for replication and growth of the parasite during bloodstage infection investigation of the interplay of iRBC and NK cells may be important to discover protective factors during the first phase of infection especially in age groups where semi-immunity has not yet developed. Several, studies showed that cross-talk between NK cells and iRBC results in activation of NK cells [10]–[12]. Therefore we address the question how NK cells sense the intracellular parasite and how they react after recognizing iRBC.

Recruitment of host-Hsp70 to the RBC membrane by *P. falciparum* was shown previously [13]. Hsp70 was demonstrated to represent a potent activator for NK cell cytotoxicity, especially when expressed on cancer cells [14]–[16]. Intracellularly, it serves as a chaperone to assist proper folding of aberrant and nascent proteins, thereby preventing apoptosis [17]. Other groups recently proposed extracellular Hsp70 as a cytokine and danger signal [7], [18]. Hsp70 was suggested to either serve as an antigen-presenting cell-activating cytokine or as a carrier for antigenic peptides to the cell surface [17]. Multhoff *et al.* discovered that a 14-amino acid oligomer (TKD peptide), localized in the C-terminal domain of Hsp70, represents an epitope recognized by activated NK cells [19]. Binding of NK cells to this epitope results in GzmB-mediated but perforin-independent apoptosis of tumor target cells [20]. Based on these findings, we addressed the questions whether iRBC express Hsp70 or other activating NK cell ligands on their cell surface, and whether iRBC are eliminated by NK cells in a GzmB-mediated manner by erythrocytic cell death. Therefore, we firstly investigated the expression of Hsp70, MICA/B, and HLA-E present on the surface of iRBC, and secondly whether the presence of one or more of these ligands impacts the expression of activating receptors such as CD94/NKG2C on NK cells. We were also interested to test whether NK cells respond to iRBC by an up-regulated expression and release of GzmB, whether perforin is involved, and finally, if NK cell activity can be further enhanced by prior stimulation with TKD and abrogated by blocking Hsp70-membrane presence.

## Results

### Co-culture of NK cells and iRBC induces growth delay of *P. falciparum*

**Figure 1:**
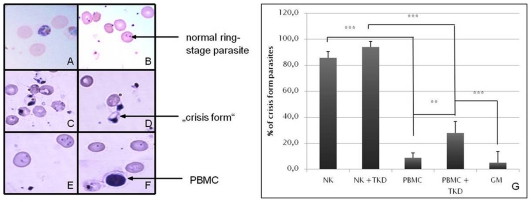
Growth delay in *Plasmodium falciparum* development after NK cell contact. A: Representative figure of 3D7-iRBC before the start of co-cultures. Parasites were synchronized by magnetic cell sorting columns for late stages. B: Representative figure of 3D7-iRBC after 24 h of incubation. The parasites have developed into normal ring-stage forms that are expected for this time point. Infected RBC were either co-cultured 3∶1 with autologous NK cells (C), NK cells+TKD (pre-stimulated for 5 days before co-culture with 2 µg/ml TKD peptide) (D), PBMCs (E), or PBMCs stimulated with TKD peptide (F). Normal parasite development of a control culture of parasites without leukocytes was observed in parallel. A blood smear was prepared before and after 24 hours of incubation and stained with 10% Giemsa. Experiments were repeated with RBC from 3 different donors.

As determined by co-culture experiments, NK cells could delay the growth or induced crisis forms of parasites, represented by packed dot-shaped forms (Figure 1C). However, in the absence of lymphocytes Plasmodia developed normally from schizonts (Figure 1A) into ring-stage trophozoites after 24 h of incubation (Figure 1B). When NK cells were pre-incubated with TKD peptide, no further visible effect was observed (Figure 1D). As shown in Figure 1E, non-stimulated PBMCs had minor influence on the development of the parasite within a 24 h co-culture period. The majority of the parasites developed normally into ring-stage parasites. Interestingly, a 5-day pre-stimulation of PBMCs with TKD peptide also induced crisis forms of Plasmodia (Figure 1F). Figure 1G shows that predominantly NK cells had an effect on parasitemia and parasite development during the 24 h co-culture period. Crisis forms were significantly induced by co-culturing iRBC with NK cells alone compared to co-culture with PBMCs (p≤0.001, student's *t* test, n = 3). A significant difference could also be detected between PBMCs (8.9±3.6%) as well as untreated iRBC (5.0±8.7%) compared to PBMCs pre-stimulated with TKD (PBMC+TKD; 27.8±8.9%, p≤0.001, student's *t* test, n = 3). The proportion of crisis forms of iRBC co-cultured with unstimulated NK cells was already very high (85.7±4.9%) and could not be significantly enhanced by using pre-activated NK cells (NK+TKD; 94.0±4.3%) (Figure 1E).

### Hsp70 but neither HLA-E nor MICA/B is present on the membrane of ring-stage infected and senescent RBC

**Figure 2:**
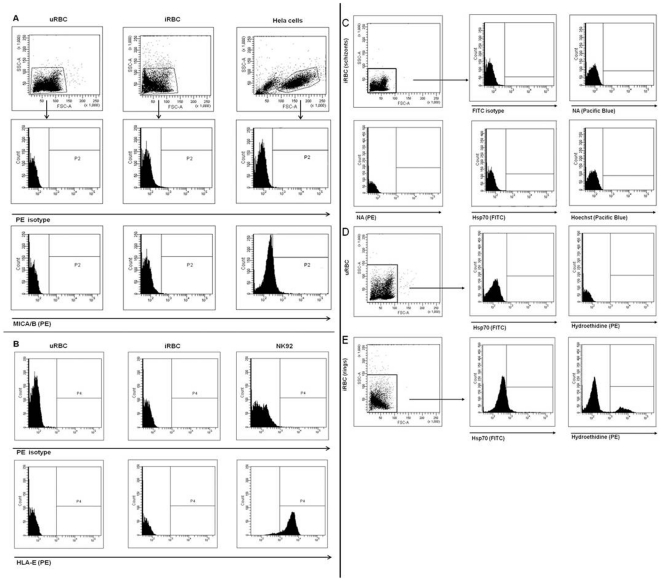
Flow cytometry analysis of erythrocytes for possible NK cell ligands. 0.5×10^6^ i/uRBC or Hela cells were stained with anti-hMICA/B-PE (A), anti-hHLA-E-PE (B) or the respective isotype control. As a control, 0.5×10^6^ iRBC, uRBC or NK92 cells were also stained with anti-hHLA-E-PE (B) or a PE-isotype control. The presence of Hsp70 on uRBC (C) and iRBC (D, E) was determined with anti-Hsp70 mAb cmHsp70.1-FITC compared to FITC isotype. Parasite DNA was stained with Hoechst dye (schizonts, D) that is detected in the Pacific blue channel or Hydroethidine (rings, E) that is detected in the PE channel. Experiments were repeated 3 times.

Since we could demonstrate that NK cells had a direct influence on parasite growth, we were interested in identifying the interaction partners of NK cells and iRBC. Therefore, the expression of Hsp70, HLA-E and MICA/B was determined by flow cytometry on iRBC and uRBC following co-culture with NK92 cells. On iRBC neither MICA/B nor HLA-E was present on the membrane (Figure 2A+B). To investigate the presence of Hsp70 on RBC, membranes of i/uRBC were stained with cmHsp70.1-FITC and analyzed by flow cytometry. Parasite DNA was stained with Hoechst or Hydroethidine to distinguish iRBC from uRBC. Hsp70 was detectable on ring-stage iRBC (stained with Hydroethidine) by flow cytometry as demonstrated in Figure 2E, but not on uRBC (Figure 2C). On schizont-iRBC (stained with Hoechst) the Hsp70 signal was not as prominent as on ring–stage iRBC (Figure 2D).

**Figure 3:**
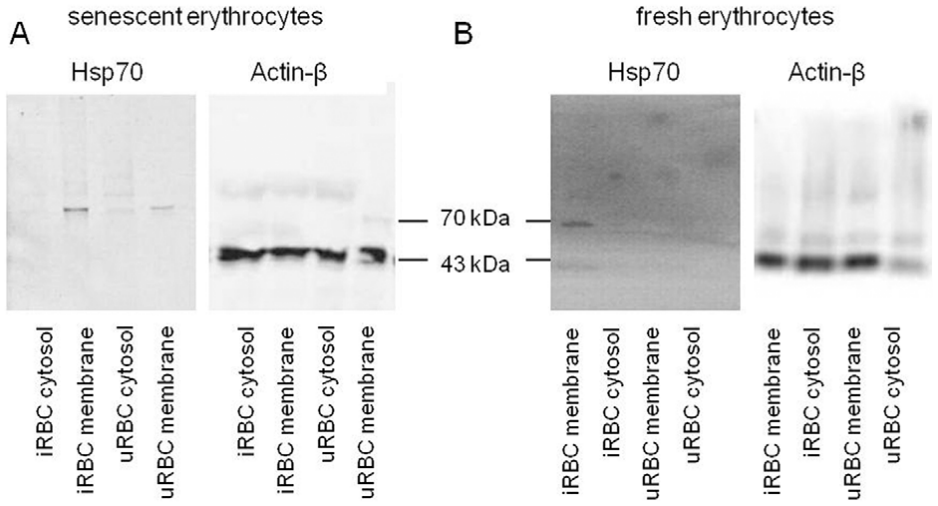
Presence of Hsp70 in the membrane of iRBC or senescent uRBC. Cytosolic and membrane protein extracts were prepared from iRBC and uRBC and submitted to SDS-PAGE. Western blots were incubated with anti-Hsp70 antibody or anti-β-Actin to control protein loading. Experiments were repeated 3 times. A: representative immunoblot of 3–4 week old erythrocytes B: representative immunoblot of fresh blood erythrocyte extracts.

To confirm these results, we assessed if host-Hsp70 is present in membrane lysates of iRBC. Protein extracts were derived from the cytosol and membrane of iRBC and uRBC. In a first attempt senescent uRBC were used. Surprisingly, Hsp70 was detectable in both infected and uninfected membrane preparations of senescent RBC (Figure 3A). When using fresh RBC only iRBC presented Hsp70 on their membrane (Figure 3B). This finding resulted in the exclusive use of fresh RBC for all other experiments.

### Characterization of cell surface markers on NK92 cells

**Figure 4:**
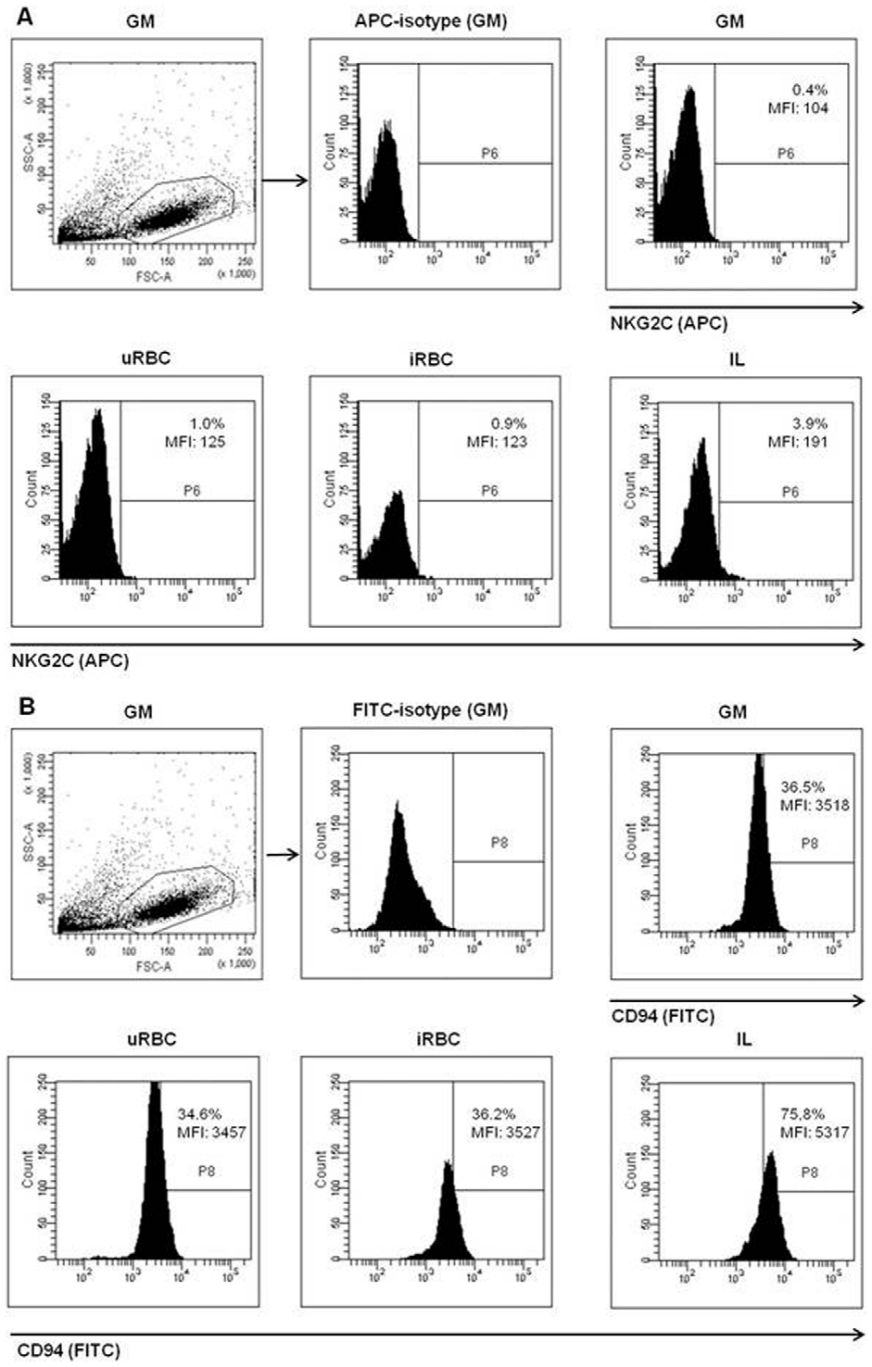
Surface expression analysis of possible recognition receptors of iRBC on NK92 cells. NK92 cells were analyzed by flow cytometry for surface expression of NKG2C and CD94. 0.5×10^6^ NK92 cells were stained with anti-CD56-FITC, anti-CD3-PE and anti-NKG2C-APC (A) or anti-CD94-FITC (B) after 24 h incubation in growth medium (GM), with 1.5×10^6^ iRBC, 1.5×10^6^ uRBC, or IL-12/-18 (IL) and analyzed by flow cytometry; a total of 10,000 events was counted for each sample. CD56^+^/CD3^−^ cells were gated according to FSC/SSC properties in the unstained autofluorescence control and compared to the respective isotype controls. Experiments were repeated three times.

In search for interaction receptors on NK cells for iRBC, the expression of surface receptors on NK92 cells was investigated. As shown, NK92 cells in the absence or presence of iRBC do express CD94 (Figure 4B) but not the activatory co-receptor NKG2C (Figure 4A) on their cell surface. Uninfected RBC had no impact on the surface expression of CD94 or NKG2C in NK92 cells. A slight up-regulation of NKG2C was observed when NK92 cells were incubated with IL-12/IL-18 (3.9% NKG2C^+^-NK92, MFI: 191) compared to cells cultured in growth medium alone (0.4% NKG2C^+^-NK92, MFI: 104). In addition, stimulation with IL-12/IL-18 also induced elevated CD94 expression compared to unstimulated cells (75.8% CD94^+^, MFI: 5317 vs. 36.5% CD94^+^, MFI: 3518).

### Cytosolic GzmB is increased on protein but not on transcriptional level in NK92 cells after contact with iRBC

**Figure 5:**
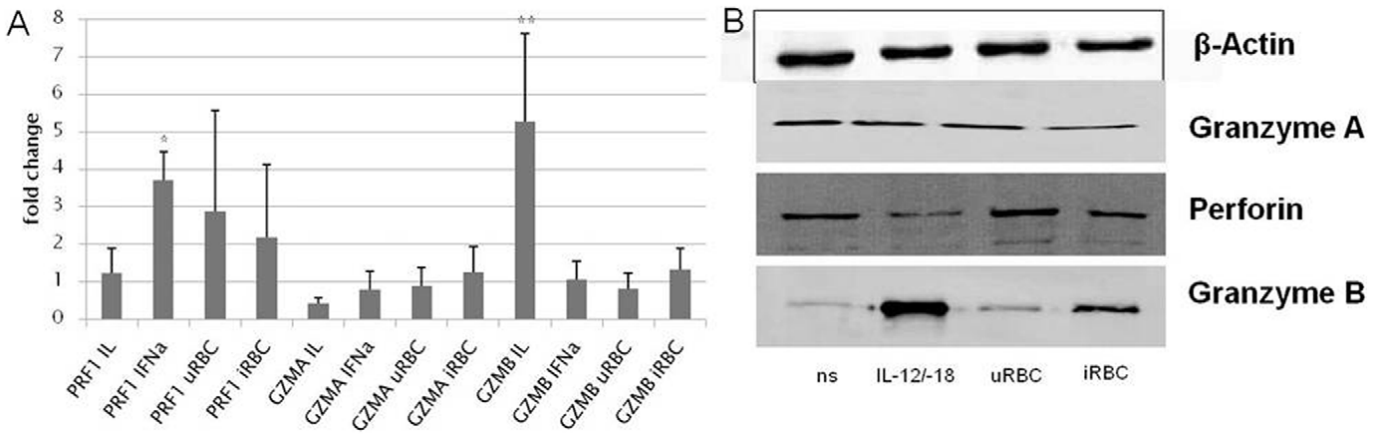
Transcriptional and translational changes of GzmA, GzmB and Perforin in NK92 cells after 24 hours stimulation. NK92 cells were left untreated (ns) or cultured with IL-12/-18 (IL), IFN-α, uRBC or iRBC (1∶3) for 24 h. A: Changes on transcriptional level of stimulated cells in comparison to untreated cells were analyzed for GzmA, GzmB and Perforin. β-Actin expression served as house-keeping gene normalizer. Data are represented as mean ± SD (student's *t* test * p<0.05; ** p<0.01) and are representative of three independent experiments each performed in duplicate. B: After 24 h, 1×10^7^ NK92 cells were lysed, incubated 30 min on ice and subsequently centrifuged at 13000× *g* for 15 min at 4°C. 10 µg of total supernatant protein were separated by 7.5% SDS-PAGE and blotted onto a nitrocellulose membrane. After blocking, membranes were incubated for 1 h with anti-β-Actin (lane 1), anti-hGzmA (lane 2), anti-hGzmB (lane 3), or anti-hPrf (lane 4).

To assess how NK cells respond to iRBC, changes in mRNA levels of GzmA, GzmB and perforin were analyzed. NK92 cells were stimulated either with IL-2/IL-12/IL-18, IFN-α or co-cultured with i/uRBC for 24 hours (1∶3). Compared to untreated NK cells, GzmB up-regulation was clearly induced when NK cells were cultured in the presence of IL-2/IL-12/IL-18 (p<0.01, student's *t* test), whereas increased expression of perforin was especially observed upon incubation with IFN-α (p<0.05, student's *t* test). Slight up-regulation of GzmB was also observed after contact of NK92 cells with iRBC (1.5-fold change). Although this effect was significantly different to that of unstimulated NK92 cells (p<0.05, student's *t* test), it cannot be considered as such since no significant differences were observed between iRBC and uRBC. None of the stimuli used did significantly affect the transcription of GzmA (Figure 5A).

To further explore effects of *P. falciparum* on the protein levels of GzmB in NK cells, cytosolic protein extracts of NK92 cells were generated after a 24 h co-culturing period with i/uRBC or following stimulation with IL-12/IL-18. Compared to unstimulated NK cells, a clear up-regulation of GzmB protein levels was detected upon stimulation of NK92 cells with IL-12/IL-18 and to a lesser extent after contact with iRBC but not with uRBC (Figure 5B). No significant influence on GzmA and perforin could be detected for the different treatments at a translational level (Figure 5B).

### Contact of NK cells with iRBC results in increased GzmB release which is inhibited by blocking Hsp70-surface expression

**Figure 6:**
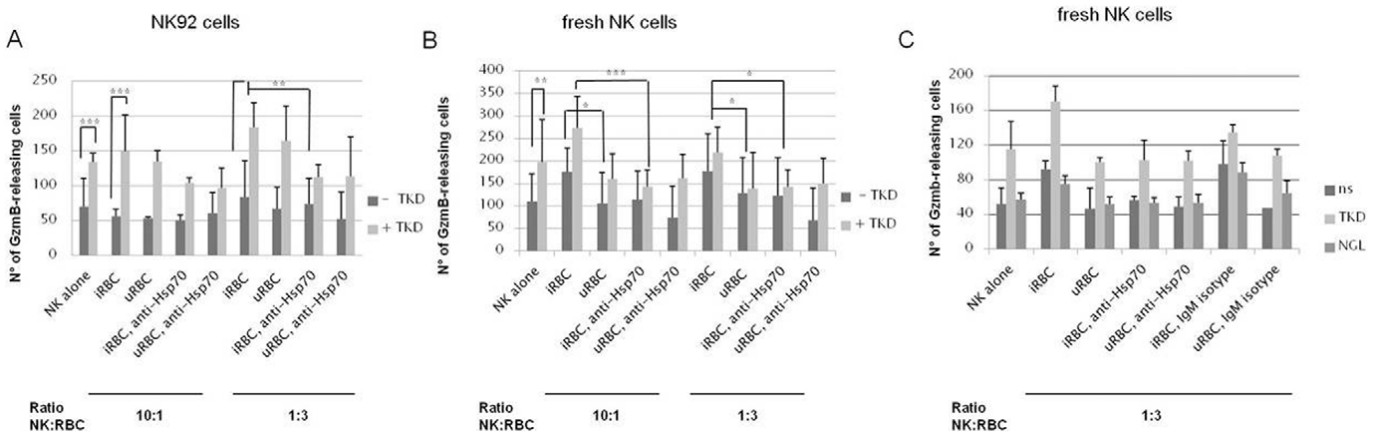
Granzyme B-Elispot of NK92 cells (A) or isolated NK cells (B,C) after 24 hours of co-culture with i/uRBC. Some cultures were pre-activated 5 days with TKD peptide or the scrambled NGL peptide and/or pre-incubated with blocking Hsp70 antibody (cmHsp70.2) or a blocking antibody IgM-isotype control for 20 minutes before the start of the experiment. After stimulation, 2000 NK cells were cultured either alone, with iRBC or uRBC (1∶3 or 10∶1) on a GzmB antibody-coated 96-well plate. GzmB-releasing cells were counted after 24 hours of incubation (* p≤0.05, ** p≤0.01, *** p≤0.001, student's *t* test, n = 6).

Considering that activated NK cells kill Hsp70 membrane positive tumor cells by GzmB-mediated apoptosis and having observed up-regulation of GzmB protein expression, we assessed the effect of parasitized erythrocytes on GzmB release of NK cells. Freshly isolated NK cells, PBMCs or NK92 cells kept for 5 days in the presence of IL-2 +/− TKD peptide were co-cultured for 24 h with i/uRBC at two different ratios, 1∶3 or 10∶1 (NK∶RBC). Previously, it was shown that NK cells exert their cytotoxic action towards Hsp70 membrane positive tumor cells in a conjoint manner (G. Multhoff, unpublished results). However, in *in vitro* culture of *P. falciparum* a ratio of 1∶3 seems to be sufficient for proper activation of NK cells. Hsp70-antibody blocking studies were performed to confirm the target specificity of the killing. In a first set of experiments, we observed that NK92 cells significantly increased GzmB release compared to untreated cells after 5 day pre-incubation with TKD peptide (p≤0.001, student's *t* test, n = 6) irrespectively of further stimuli such as iRBC or uRBC (Figure 6A). Similar results were obtained for primary NK cells (Figure 6B). However, the difference of NK92 cells co-cultured with iRBC was not significantly altered in comparison to untreated cells. No significant differences in GzmB release were seen with PBMCs after an identical treatment regimen (data not shown). However, if experiments were repeated using freshly separated NK cells, significant differences could be detected (Figure 6B). Due to the allogenicity of the NK/RBC system, a basal release of GzmB by NK cells was observed, irrespectively of the co-cultivation with uRBC or iRBC (data not shown). In an autologous system, only iRBC led to a significant increase in GzmB release by autologous NK cells at both ratios compared to uRBC (p≤0.05, student's *t* test). GzmB secretion could be completely blocked down to basal levels after the addition of anti-Hsp70 blocking antibody at a ratio of 1∶3 (NK∶iRBC) for NK cells that were co-cultured with iRBC (p≤0.05, student's *t* test). The effect of TKD+iRBC was especially reversed by the anti-Hsp70 blocking antibody at a ratio of 10∶1 (p≤0.001, student's *t* test). The specificity of the effect was verified with another set of experiments where additionally freshly isolated NK cells were pre-incubated with the scrambled NGL peptide (Figure 6C). No difference to untreated NK cells was observed. Furthermore, the specific action of the Hsp70 blocking antibody was evaluated by addition of an IgM isotype control 20 minutes prior to co-culture with u/iRBC (Figure 6C). Only when NK cells were pre-activated with TKD peptide and afterwards co-cultured with iRBC, did addition of the IgM isotype have a significant decreasing effect (student's *t* test, p≤0.05). However, the amount of GzmB-releasing cells was still significantly higher in comparison to TKD-pre-stimulated NK cells that were co-cultured with cmHsp70.2-treated iRBC (student's *t* test, p≤0.05). If no pre-stimulation was applied the abrogating effect of cmHsp70.2 was even more pronounced (iRBC+blocking mAb (ns) vs iRBC+IgM isotype (ns): student's *t* test, p≤0.01).

In summary, NK92 cells as well as freshly isolated NK cells displayed a significantly increased release of GzmB 5 days after stimulation with TKD peptide. Co-culturing of NK cells with iRBC, but not uRBC, further increased the release of GzmB. The addition of Hsp70 blocking antibody specifically abrogated the stimulatory effect induced by TKD and iRBC.

### NK cells induce eryptosis of iRBC

**Figure 7:**
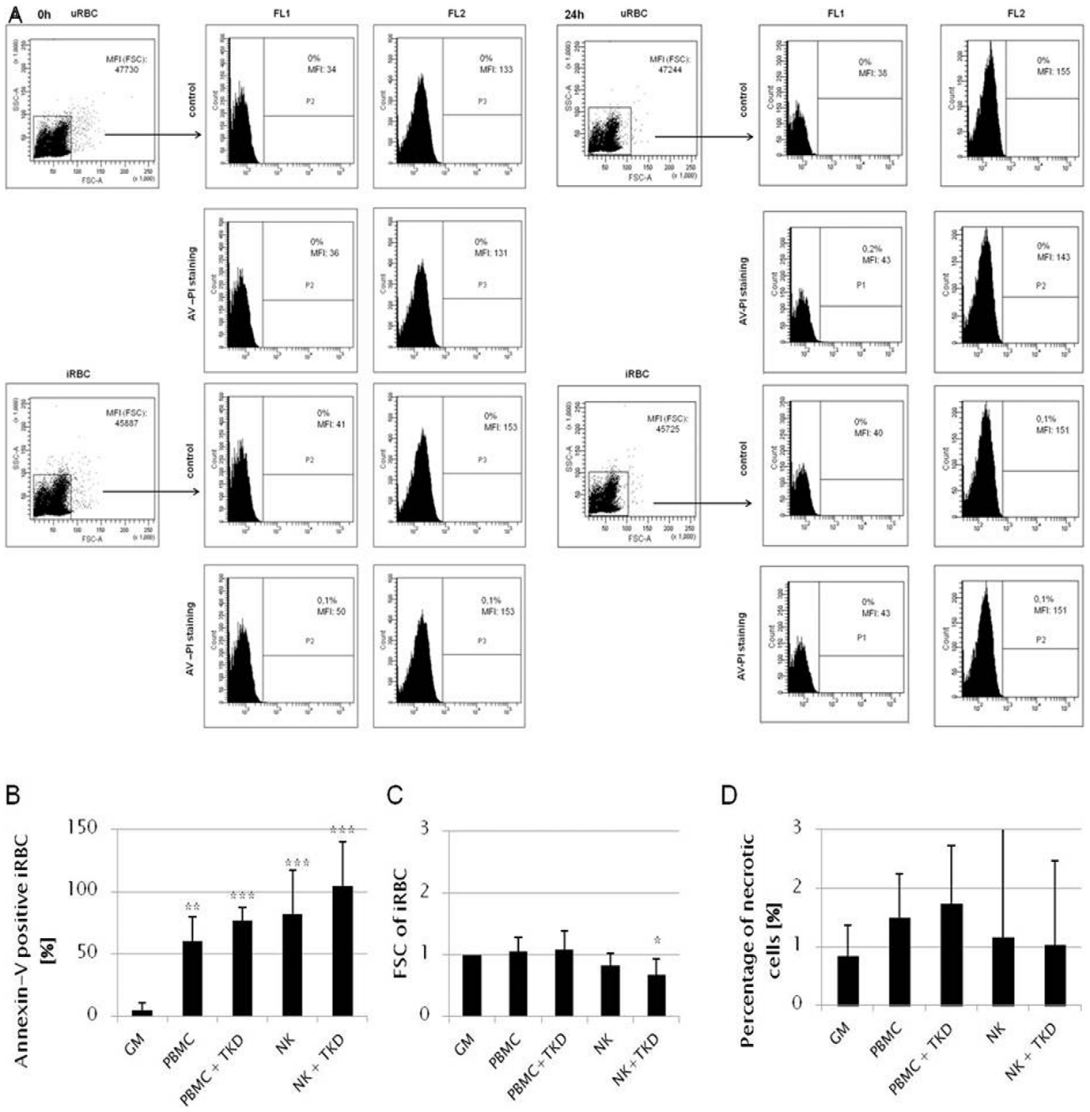
Eryptosis of iRBC after co-culture with PBMCs or purified NK cells. iRBC were co-cultured 3∶1 for 24 hours with NK cells, NK cells+TKD (5 day stimulation), PBMC, PBMC+TKD (5 day stimulation) of the same donor. 0.5×10^6^ erythrocytes were washed in 5 mM Ringer solution. Afterwards, cells were stained for 15 minutes with Annexin-V (1∶500) and propidium iodide (1∶50). Eryptotic cells were determined as Annexin-V-positive (AV^+^) and propidium iodide-negative (PI^−^). A: Baseline levels of eryptotic uRBC and iRBC at the start of the experiment and after 24 h of culture in growth medium without leukocytes. RBC were stained with AV+PI or left unstained. Gating was done based on FSC/SSC properties and the unstained control. AV was measured in the FL1 channel and PI in the FL2 channel. B: Percentage of AV^+^/PI^−^-iRBC after co-culture in growth medium (GM) or with different effector cells in relation to starting parasitemia (** p≤0.01, student's *t* test, n = 6). C: Normalized FSC of iRBC after co-culture in growth medium (GM) or with different effector cells (* p≤0.05, student's *t* test, n = 6). FSC values of co-cultured iRBC were normalized to untreated iRBC (GM). D: PI^+^ necrotic iRBC after culture in growth medium (GM) or with various effector cells. Numbers of necrotic cells were normalized to untreated iRBC (GM).

Since we could show that Hsp70 membrane expression as well as GzmB release are important characteristics of the responses of NK cells towards iRBC, we were interested whether cell death is induced. Programmed death of iRBC was evaluated after 24 h of co-culture with either autologous PBMCs or NK cells that were pre-stimulated with/without TKD. Necrotic cells were excluded by propidium iodide staining. No spontaneous eryptosis of uRBC or iRBC was observed at the beginning of the experiment as well as 24 h later (Figure 7A). Our results demonstrate that iRBC undergo eryptosis but not necrosis following contact with NK cells (NK vs GM: p≤0.001). This effect was even more pronounced when TKD-stimulated NK cells were used (NK vs NK+TKD: p≤0.01). Also PBMCs had the capacity to induce eryptosis of iRBC, however, at a lower level (Figure 7B, p≤0.01). Signs of eryptosis could also be detected by smaller cell size compared to untreated iRBC measured as reduced FSC properties of erythrocytes after co-culturing with NK cells (Figure 7C). Again, stimulation with TKD peptide enhanced the effect significantly (n = 6, p≤0.05). Addition of PBMCs or PBMCs stimulated with TKD did not significantly alter cell shrinkage (Figure 7C). The number of necrotic iRBC was always low and ranged between 0.8±0.8% in growth medium to 1.7±2.0% after co-culture with PBMCs+TKD (Figure 7D).

## Discussion

**Figure 8:**
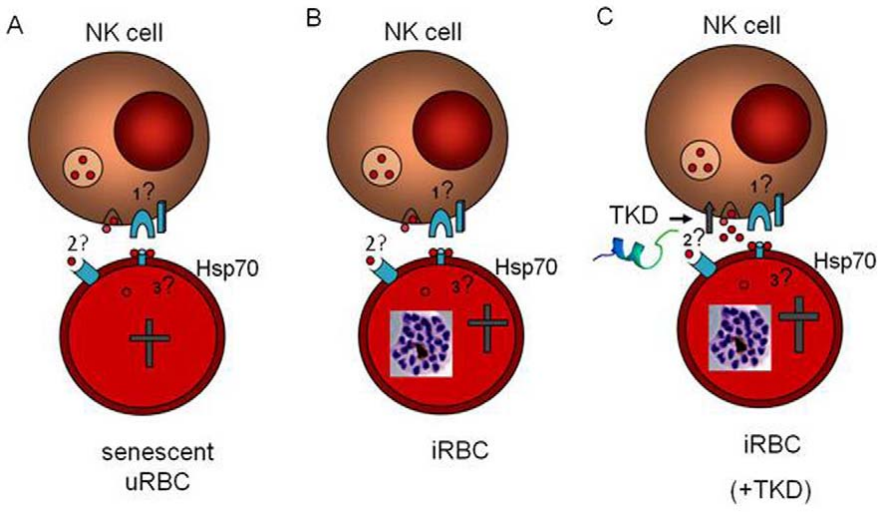
Hypothetical model of NK cell response to iRBC and senescent uRBC. If erythrocytes become senescent (A) or are infected with *Plasmodium falciparum* (B,C) host-Hsp70 will be recruited to the cell membrane. ?1 NK cells recognize Hsp70-exposure by yet unknown receptors, possibly CD94. Recognition of Hsp70 leads to GzmB release. ?2 GzmB will enter the target cell with either assistance of Hsp70, an unknown receptor or become endocytosed. ?3 Once inside the iRBC, GzmB induces eryptosis. Pre-stimulation with TKD peptide enhances both GzmB release and the amount of eryptotic iRBC (C).

In the present study, we investigated a new hypothetical pathway by which senescent (Fig. 8A) or iRBC (Fig. 8B) are recognized and marked for removal by NK cells. This model implies that upon infection with *P. falciparum* or when aged erythrocytes display Hsp70 on their surface. This results in GzmB release probably triggered by a yet unknown NK cell receptor. Pre-stimulation of effector cells with Hsp70-peptide TKD leads to an increased release of GzmB. Subsequently, GzmB will either become endocytosed, be up-taken with the help of an unknown receptor or concomitantly by internalization of Hsp70 by RBCs which finally will undergo eryptosis.

First insights into the investigated interaction were obtained by co-culture experiments. We have previously found that direct impact of NK92 cells on iRBC results in reduced parasitemia after 48 h of co-culture [21]. Additionally, we observed that NK92 cells suppress the expression of vital genes in the *P. falciparum* laboratory strain FCR3-CSA (unpublished results). However, in these experiments we could not exclude that those effects were a sign of nutrient depletion in the culture system. In the present study, we show that the observed impairment of parasite development is directly mediated by NK cells. The induction of parasite crisis form was much more pronounced in co-cultures of fresh iRBC with isolated NK cells compared to those where PBMCs were co-cultured. Because NK cells represent only 5–15% of PBMCs it seems that the number of NK cells within the PBMC fraction may be too small to efficiently affect the parasite in the iRBC. However, when PBMCs were pretreated with TKD we also found a significant increase of crisis forms within the iRBC compared to PBMCs. This could be explained either by the fact that within the PBMC fraction also other effector populations than NK cells will be activated by TKD and affect the parasites or that NK cell activation is more efficient in the presence of other immune cells upon stimulation with TKD.

That pre-treatment of lymphocytes with the Hsp70 TKD peptide had a significant impact on parasite development suggests that iRBC serve as targets for NK cells through their membrane expression of Hsp70. The origin of membrane Hsp70 remains unclear. It was only detected in small quantities in the cytosol of senescent but not of fresh RBC, although RBC do not have the protein machinery for *de novo* synthesis. Other authors could show presence of Hsp70 in the RBC cytosol [13] possibly because Hsp70 is a well-known chaperone residing in the cytosol. The reason that we could not detect sufficient cytosolic amounts might be due to antibody sensitivity as a long visualization time was required in order to detect Hsp70. Interestingly, we also found Hsp70 expression in senescent erythrocyte membranes. This suggests that senescent erythrocytes might also be targets for the cytolytic attack of NK cells. Up to now it remained enigmatic why MHC-negative erythrocytes are not target cells for the cytolysis of NK cells. We suggest that missing-self [10] is not sufficient to activate NK cells and assume that additional activatory signals are required to induce killing activity of NK cells. We could identify that membrane-expressed Hsp70, selectively expressed on iRBC but not on uRBC, acts as a key stimulatory factor for NK cell activation. However, if host-Hsp70 membrane exposure on iRBC has negative or beneficial effects for parasite survival *in vivo* remains to be elucidated. Perhaps Hsp70 is involved in the formation of crucial parasite-derived protein complexes at the RBC surface that are required for cytoadherence or immune evasion. As suggested by others, it may serve to transform the RBC membrane [22]. This seems likely since Hsp70 was only present in early but not late stages of parasite development when RBC membrane remodeling events mainly take place [23], [24]. Hsp70 is expressed on senescent erythrocytes as well. Therefore, Hsp70 might represent a physiological removal sign such as aggregated band 3, which is formed rapidly during parasite growth within the iRBC [25]. On the other hand, Hsp70 could act as a danger signal or exert chaperone function by transporting immunogenic peptides to the surface of iRBC in order to alert the immune system to clear the parasite infection. The knowledge of potential antigens that are presented with the help of Hsp70 would therefore deepen our understanding of the complete mechanism involved.

Apart from Hsp70, we aimed to investigate additional ligands on iRBC and their corresponding receptors on NK cells. The balance of the presence and absence of Hsp70 and HLA-E were reported to be relevant for NK cell cytotoxicity [8], [26]. Hence, HLA-E expression was analyzed on iRBC as well as presence of its receptor NKG2C on NK cells. Co-culture of NK92 cells with iRBC results in an up-regulation of NKG2C as previously determined in microarray assays [21]. However, in our experiments, neither MICA/B nor HLA-E was found to be present on the surface of iRBC. The co-receptor CD94 but not NKG2C was present on NK cells irrespectively of parasitic infection of RBC meaning that CD94 could be involved in the NK cell erythrocyte interaction. The expression density of CD94 is important for the cross-talk of Hsp70 membrane-positive tumor cells with NK cells and it was formerly reported to lead to higher anti-Hsp70-activity [27]. Moreover, NK cells with an elevated CD94/NKG2 expression were previously reported to produce high amounts of IFN-y [12]. In the present study we have not identified whether CD94 is the interaction partner of Hsp70 or not and further studies have to be performed to solve this question. Human mature NK cells are heterogeneous for the expression of numerous receptors (NKG2 family, KIR family) or maturation markers such as CD57 and CD62L. Thus, detailed investigation of resting NK cells are required to evaluate the role of these different subsets in Hsp70-mediated recognition of iRBC.

In the context of malaria an involvement of accessory cells was assumed. In these studies activation of NK cells was mainly shown through their expression of CD25, CD69 and the production of IFN-γ [2], [10]. Our findings support the importance of cytokines derived from accessory cells for an optimal stimulation of NK cells. Here we extend the role of IL-12/-IL-18 to trigger cytotoxicity via up-regulation of GzmB transcription and its cytosolic abundance. In our experiments iRBC enhanced cytosolic expression and release of GzmB, possibly mediated via the presence of Hsp70 as shown by Hsp70 blocking experiments, where GzmB release was inversed. This effect was also observed to a lesser extent using an isotype control. This suggests that other stress signals and/or activating NK receptors such as DNAM-1, NKp30 or NKp46 might be additionally involved in this process. The finding that only translation but not transcription of GzmB was up-regulated is in accordance with other investigations. High levels of mRNA but low protein expression of GzmB and perforin in resting NK cells or CTL but elevated protein expression after activation were observed in different T-cell subsets and NK cells [28], [29]. Furthermore, a pre-existing pool of mRNA of GzmB and perforin that can be quickly translated into protein after stimulation was reported from a mice study [30]. In addition, translational repression of GzmB/perforin by micro-RNA was recently demonstrated in human NK cells [31]. Thus, the result of elevated GzmB protein expression after co-culture implies that through iRBC contact translation might be activated. GzmB release in response to iRBC could be further raised by prior sensitization of NK cells with the Hsp70 TKD peptide. Whether such enhancement is beneficial or harmful for the host requires to be elucidated. It was previously shown that GzmB levels are elevated in individuals with severe malaria [32]. However, it remains unclear whether this is cause or result of the disease. In other studies, up-regulation of perforin and GzmA but not GzmB was detected in NK cells following PBMC co-culture with iRBC. The reason why we could not detect perforin or GzmA might be due to the fact that we were using isolated NK cells meaning that in other settings perforin or GzmA might be released because of accessory cells. Additionally, the fact that NK92 are transformed cells that already underwent differentiation also needs to be taken into account.

Recently it was shown that Hsp70 recognition by NK cells leads to apoptosis of tumor cells in a GzmB-dependent but perforin-independent manner [20]. Erythrocytes cannot undergo classical apoptosis characterized by DNA degradation since they are non-nucleated, highly specialized cells whose main function is oxygen transport. Nevertheless, many studies have observed a form of programmed cell death called eryptosis, which is characterized by membrane scrambling and cell shrinkage [33]. These authors also showed that eryptosis occurs during infection of erythrocytes with *P. falciparum* [34]. The mechanism, however, was not yet characterized. We could show that eryptosis was induced by co-culturing iRBC with different effector lymphocytes. In our experiments, we excluded spontaneous eryptosis of u/iRBC cultured without lymphocytes showing that eryptosis depends on cytotoxic cells which are co-cultured with the erythrocytes. Since parasitemia at the beginning of the co-culture varied from 2.1–19.0% the number of eryptotic iRBC varied as well in each experiment suggesting that only infected erythrocytes are affected by eryptosis. Furthermore, we could also detect eryptosis in iRBC co-cultured with PBMCs, which suggests that either other lymphocytes (e.g. CD8^+^ T cells) may also be activated to elicit cytotoxicity or that a larger proportion of NK cells are properly activated due to signals delivered by other PBMCs.

If supported by further studies, our results could provide the basis for a new strategy to treat malaria. Our experiments show that NK cells as well as TKD-pretreated cells have an anti-parasitic effect but we do not know if this treatment completely clears the infection and more importantly, will prevent future infections with the parasite. Nevertheless, it is important to identify and to evaluate basic mechanisms occurring during *P. falciparum* infection in order to find adjuvants or drugs which act on specific cell types and to develop feasible applications for future settings. Feasibility, safety and toxicity of adoptively transferred *ex vivo* TKD-stimulated autologous NK cells were already proven in a phase I clinical trial in lung and colon cancer patients [35]. Several clinical trials with different types of cancers employing heat shock protein based-vaccines were already undertaken [36]. Furthermore, Hsp70-peptide complexes (Hsp70-PC) can also activate other immune cells such as antigen presenting cells (e.g. DCs), leading to maturation via NF-kB-activation. This activation results in stimulation of cytokine release (TNF-α, IL-1b and IL-6), expression of co-stimulatory molecules (B7.1, B7.2, CD40 und MHC class-II) as well as release of nitric oxide. Additionally, APCs are known to internalize Hsp70-PC and then present them as MHC-complexes which involve both the innate and the adaptive immune response [37]. All these mechanisms play a role in malaria for which reason it is conceivable that Hsp70 is important in this context. Apparently, NK cell activation by TKD peptide is not easily applicable in malaria, first, because in contrast to cancer, malaria is an acute disease which requires an immediate treatment. Moreover, it is at the moment unlikely to perform *ex vivo* TKD-stimulated NK cell treatment that requires cost intensive GMP-facilities in resource-poor countries where the burden of malaria is highest. However, one could imagine directly injecting the peptide into patients in order to stimulate NK cells, *in vivo*. Recently, several studies have proposed that NK cells have a memory [38], [39]. If this is the case, administration of TKD peptide, maybe in combination with a standard vaccine would help to better respond to Hsp70-positive iRBC during *Plasmodium* infection and also to other modified cells in diseases like virus infection or cancer. How long does this effect last? How can we assure that the TKD peptide reaches NK cells in appropriate concentrations? Many issues remain to be solved. Nevertheless, our proposed model opens the way for new alternatives, which are urgently needed.

## Methods

### Ethics statement

Freshly isolated PBMCs and NK cells were obtained from six healthy malaria-naïve donors after written informed consent. The study was approved by the Ethical Committee of the Medical Faculty of Tuebingen University and the University Hospital of Tuebingen.

### Cell line

NK92 cells (DSMZ) were grown at 0.2–0.6×10^6^ cells/ml in 75% α-MEM, 12.5% fetal bovine serum (FBS) and 12.5% horse serum supplemented with 2 mM L-glutamine, 10 ml/l penicillin – streptomycin, 10 ng/ml recombinant human interleukin-2 (rIL-2). *Mycoplasma* contamination was prevented by addition of 5 µg/ml Plasmocin and routinely excluded by PCR.

### PBMC and NK Cell isolation

Venous blood was collected into ammonium heparin tubes (10 IU/ml blood, Sarstedt GmbH, Nuembrecht, Germany), isolated by Ficoll separation (GE Healthcare), washed twice with 2% FBS in RPMI and resuspended at 2.5–5×10^6^ cells/ml in RPMI supplemented with 6 mM L-glutamine, 5% FBS, 100 U/ml penicillin-streptomycin and 100 U/ml IL-2 (Invitrogen). NK cells were isolated with Dynabeads® Untouched™ Human NK Cells Isolation Kit (Dynal Invitrogen, Oslo, Norway), washed twice with isolation buffer (2% FBS in phosphate buffered saline (PBS) supplemented with 2 mM EDTA) and cultured in 24-well plates at a density of 2×10^6^ cells/ml. Purity of NK cells (≥93.5%) was verified by CD3-PE/CD56-FITC staining (Becton Dickinson).

### *P. falciparum* culture

*P. falciparum* parasites (strain 3D7) were maintained in continuous culture as described elsewhere [40]. Parasites were either grown in 0^Rh+^ human erythrocytes (Blood bank, University Hospital Tübingen, Germany) or in autologous RBC from the respective donor in RPMI 1640 supplemented with 25 mM HEPES (Sigma-Aldrich), L-glutamine (PAA), gentamycin and 50 ml Albumax II. Mature schizont-infected erythrocytes were harvested by magnetic cell sorting LD separation columns (MACS; Miltenyi Biotec). Parasite cultures were routinely screened for *Mycoplasma* contamination by PCR.

### NK cell stimulation

To prevent prior activation, NK92 cells were kept in culture medium in the absence of rIL-2 overnight. NK92 or freshly isolated NK cells were either co-cultured with iRBC, uRBC (1∶3) or stimulated with IL-12/IL-18 (100 ng/ml each) or IFN-α (100 U/ml) for 24 h at 37°C and 5% CO_2_. Some cultures were stimulated five days prior to co-culture with 2 µg/ml TKD (multimmune GmbH, Munich, Germany) peptide (purity>96%; Bachem, Bubendorf, Switzerland), since it was previously demonstrated that this period is optimal for activating NK cells towards Hsp70^+^ target cells [8], [35].

### Growth delay

To evaluate the effect of freshly isolated NK cells on parasite growth, NK cells or PBMCs were pre-stimulated with/without TKD for 5 days prior to experiment and co-cultured with iRBC (1∶3) from the same donor. After 24 hours, blood smears were prepared from the pellets. Cells were fixed in methanol and subjected to 10% Giemsa staining for 20 minutes. Development of parasites was evaluated in comparison to untreated iRBC before and after co-culture. Photography was taken at 1000× amplification.

### Cell surface staining for flow cytometry to detect possible interaction partners on the NK cell and iRBC surface

NK cells were characterized by flow cytometry using fluorescence labelled CD56 (FITC, BD Biosciences), CD3 (PE, BD Biosciences), CD94 (FITC, R&D Systems) or NKG-2C (APC, R&D Systems) antibodies. Both uRBC and iRBC were incubated with anti-hMICA (PE, R&D Systems), anti-hMICB (mouse monoclonal IgG_2B_, clone 236511, R&D Systems), subsequently stained with goat anti-mouse IgG_2B_-FITC (1 µg IgG_2B_/10^6^ cells, Santa Cruz), hMICA/B (PE, clone 6D4, eBioscience), or anti-hHLA-E (PE, eBiosciences). The presence of Hsp70 on RBC was determined with anti-Hsp70 (Alexa 488-conjugated, clone D69, Cell Signaling) or cmHsp70.1 (FITC, IgG_1_, multimmune GmbH, Munich, Germany). Parasite DNA was stained with Hoechst dye for 30 minutes at 37°C in the dark or with Hydroethidine (0.014 mg/ml; Polysciences, Eppelheim, Germany). Dead cells were excluded by 7-AAD staining (BD). Flow cytometry measurements were carried out using a FACSCanto flow cytometer (BD Biosciences) and analyzed with BD FACS Diva 6.0 software. Gating was done based on forward and sideward scatter properties.

### Real-time quantitative analysis of GzmB, GzmA, and Perforin expression

Total RNA was extracted from 3×10^6^ NK92 cells with RNeasy MiniKit (Qiagen, Hildesheim, Germany). 0.5 µg RNA was converted into cDNA using Quantitect Reverse Transcription Kit (Qiagen, Hildesheim, Germany). For real-time PCR the following Quantitect Primer Assays (Qiagen, Hildesheim, Germany) were used: QT01001875 Hs_GZMB_2_SG (GzmB), QT00015575 Hs_GZMA_1_SG (GzmA), QT01869602 Hs_PRF1_2_SG (Perforin), and QT01680476 Hs_ACTB_2_SG (β-Actin). The reaction mix was prepared according to the standard protocol of Rotor-Gene SYBR Green RT-PCR Kit (Qiagen). RT-PCR was carried out in triplicate with a Rotor Gene 3000 Cycler with standard thermal profile. Transcriptional changes were calculated with the ΔΔCt-method, excluding Ct-values≥35 and applying a threshold of 0.01.

### Western Blot

1×10^7^ cells were lysed in 1 ml lysis buffer (20 mM HEPES, 250 mM NaCl, 20% glycerine, 1 mM MgCl_2_, 0.5 mM EDTA, 0.1 mM EGTA, 1% Np-40 (IGEPAL), 1 mM Dithiothreitol, and proteinase inhibitor (1 tablet complete protease inhibitor / 50 ml), incubated 30 min on ice and subsequently centrifuged at 13000× *g* for 15 min at 4°C. Supernatants containing the RBC cytosol were removed, pellets with the RBC membranes were washed twice with lysis buffer and the supernatants were combined. 10 µg of total protein were boiled at 95°C for 10 min in reducing loading buffer, separated by 7.5% SDS-PAGE and blotted 45 min onto a nitrocellulose membrane (Bio-Rad). Blocking was performed overnight with 4% bovine serum albumin (BSA) in 1% PBS-Tween-20 (PBST), membranes incubated for 1 h with either anti-hGzmA (GA6, Santa Cruz, 0.5 µg/ml), anti-hGzmB (Santa Cruz, 0.1 g/ml) or anti-hPrf (4E4, Santa Cruz, 0.5 µg/ml). Separated and blotted RBC extracts were incubated with anti-Hsp70 (D69, Cell Signaling, 1∶300) and subsequently incubated with rabbit anti-mouse IgG linked to horseradish peroxidase (Cell Signalling, 1∶3000). Conjugated proteins were visualized with ECL reagent (Amersham Corp.) according to manufacturer's instructions. For control of equal protein loading, membranes were incubated in stripping buffer (62.5 mM Tris-HCl pH 6.7, 100 mM β-mercaptoethanol, 2% SDS) at 50°C for 30 min under occasional agitation. Following washing with PBST, blocking with BSA-PBST was repeated as described above and membranes were incubated with mouse anti-hActin (0.5 µg/ml ACTBD11B7, Santa Cruz), followed by immunoblot as described before.

### Determination of release of cytotoxic effector molecules using GzmB ELISPOT assay

A non-radioactive assay was chosen to assess GzmB release of freshly isolated NK cells or NK92 cells cultured with rIL-2 and with/without TKD peptide (2 µg/ml) or a 14-mer scrambled NGL peptide (NGLTLKNDFSRLEG) consisting of the same amino acid residues in a different order (2 µg/ml; Bachem, Bubendorf, Switzerland) for 4–5 days prior stimulation with i/uRBC (1∶3 or 10∶1). The same set of experiments was repeated using a blocking antibody directed against membrane Hsp70 by pre-incubating i/uRBC with 10 µg/ml cmHsp70.2 (IgM, multimmune GmbH, Munich, Germany) for 20 minutes or a control IgM isotype (clone MM-30, 10 µg/ml, Biolegend). Granzyme B release was elaborated using ELISPOT assays (Abcam, Cambridge, UK) following the standard protocol with 2000 effector cells (ratio 1∶3 or 10∶1 of NK cell to u/iRBC) in complete RPMI for 24 hours at 37°C. Spot formation was monitored and counted using an ImmunoSpot Series 5.0.9 Analyzer (CTL-Europe GmbH, Aalen, Germany).

### Eryptosis assay

Previous reports showed eryptosis, an apoptosis-like cell death of erythrocytes triggered by a broad variety of stimulators such as temperature increase [41], and others [33]. To determine whether eryptosis of iRBC is induced due to NK cell contact, phosphatidylserine exposure was assessed by measuring Annexin-V-staining at an excitation wavelength of 488 nm and propidium iodide (PI) at an excitation wavelength above 600 nm; in addition forward scatter (FSC) was recorded. After 24 hours of co-culture with PBMCs or isolated NK cells (3∶1) that were sometimes pre-incubated 5 days with TKD peptide (2 µg/ml) before co-culture, 0.5×10^6^ erythrocytes were washed in 5 mM Ringer solution (NaCl, KCl, CaCl, HEPES, glucose). Afterwards, cells were stained for 15 minutes with Annexin V-FLUOS (1∶500, Roche, Mannheim, Germany) and PI (1∶50).

### Statistical analysis

Statistical analysis was performed using JMP for Windows 5.0.1 (SAS Institute Inc., Cary, North Carolina), applying either student's *t* test, assuming unequal variances, or one-way ANOVA. Results were considered statistically significant at *Ρ*<0.05.
